# Supplementary material for: Mechanism of substrate hydrolysis by the human nucleotide pool sanitiser DNPH1
Source: Nat Commun. 2023 Oct 26;14:6809. doi: 10.1038/s41467-023-42544-4 (PMC10603095; doi:10.1038/s41467-023-42544-4)
Supplement: Supplementary file 1 — Supplementary Information [file 41467_2023_42544_MOESM1_ESM.pdf]

Supplementary Information

Title: Mechanism of substrate hydrolysis by the human nucleotide pool sanitiser DNPH1

Authors: Neil J. Rzechorzek, Simone Kunzelmann, Andrew G. Purkiss, Mariana Silva Dos Santos, James I. MacRae, Ian A. Taylor, Kasper Fugger and Stephen C. West

Includes:

6 Supplementary Figure

3 Supplementary Tables

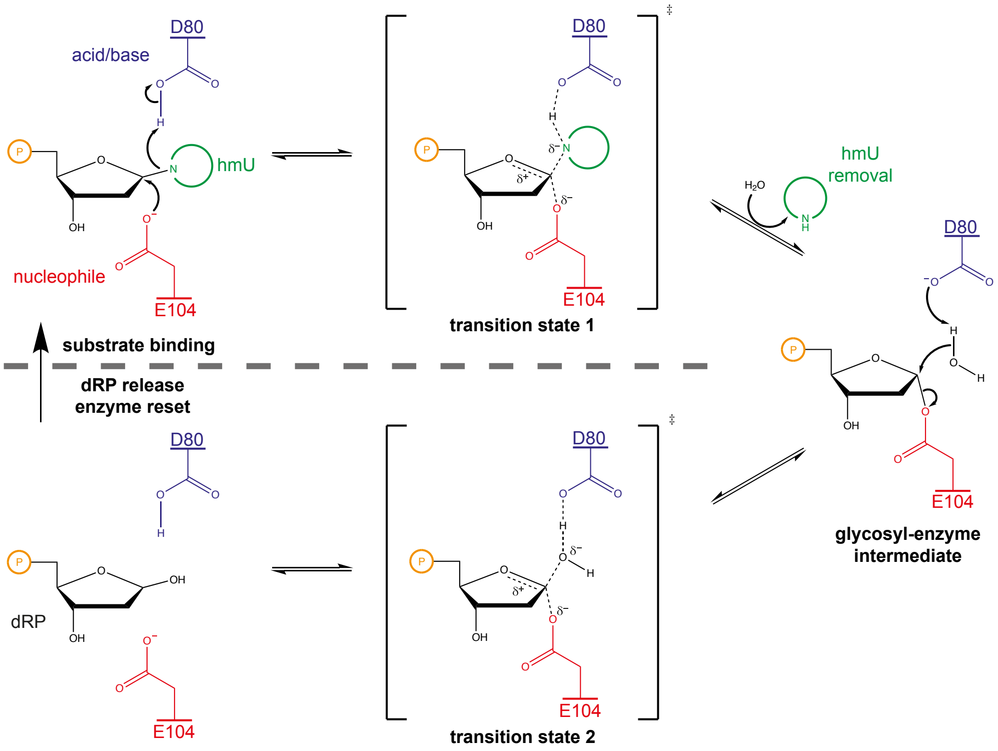

Supplementary Fig. 1: Two-step reaction mechanism for retaining N-glycosidases. These enzymes utilise two conserved carboxylate groups: a nucleophilic glutamate (red) that generates a glycosyl-enzyme intermediate, and an aspartate (blue) that protonates the hmU leaving group during the first step and activates the nucleophilic water during the second. Substrate phosphate, deoxyribose and hmU moieties are coloured orange, black and green respectively. Carboxylate sidechains shown from Cβ. Residue identities relate to DNPH1.

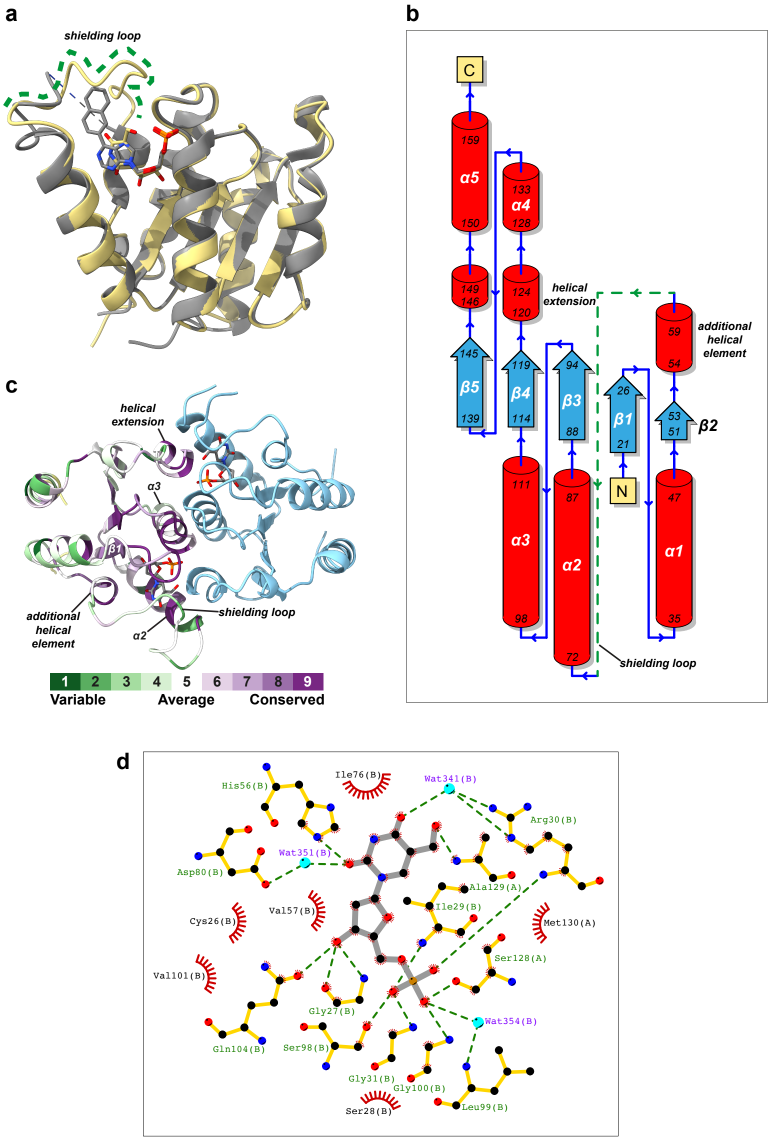

Supplementary Fig. 2: Global and active site architectures of DNPH1. a, Superposition of DNPH1^{E104Q} chain B bound to hmdUMP (yellow) with chain A of DNPH1 bound to a purine-based inhibitor (grey; PDB 4P5E) RMSD between 128 Cα atom pairs = 0.413 Å. **b**, Topology diagram of DNPH1^{E104Q}, with sequentially labelled blue β-sheets (β1-β5) and red α-helices (α1-α5). Numbers refer to amino acid boundaries of secondary structure elements. Additional helical element and helical extension, along with N- and C-termini, are shown. The shielding loop is indicated by green dotted line. **c**, DNPH1^{E104Q}, as in (Fig. 2a), with chain B coloured according to conservation. Active site cleft (formed by β1, α2 and α3), shielding loop and key helical moieties are labelled. Regions of chain B with insufficient conservation data are coloured yellow. **d**, 2D schematic of ligand interactions in the DNPH1 active site. hmdUMP and protein side chains involved in hydrogen bonds (green dotted lines) are shown as grey and yellow sticks, respectively. Carbon, oxygen, nitrogen and phosphorus atoms are coloured black, red, blue and orange, respectively. Interacting water molecules are shown as light blue spheres. Hydrophobic interactions represented as red arcs. In a and c, ligand/sidechain oxygen, nitrogen and phosphorus atoms coloured red, blue and orange respectively.

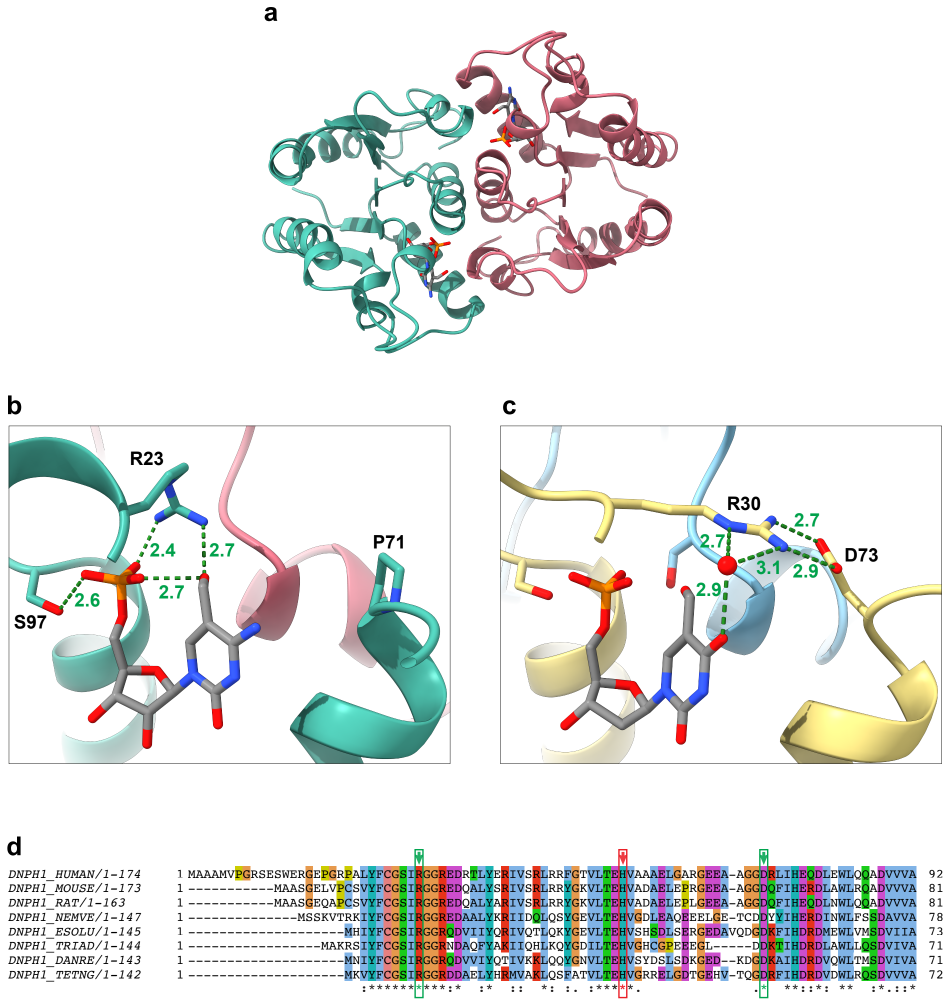

Supplementary Fig. 3: DNPH1 shares similarities and differences with bacterial MilB. a, Cartoon representation of dimeric MilB^{E103A} (PDB: 4OHB) bound to hmCMP (grey sticks). Biological dimer generated by crystallographic symmetry; symmetry-related chains are coloured green and red. **b**, Close-up view of hmCMP (grey sticks) bound to the active site of MilB (green), highlighting the lack of stabilisation by the adjacent subunit (red). Substrate phosphate and hydroxymethyl moieties are instead positioned by conserved R23. **c**, Close-up view of hmdUMP (grey sticks) bound to the active site of DNPH1^{E104Q} (yellow, blue), viewed as in (b). The base is stabilised by a water (red sphere) positioned by conserved R30; the latter is stabilised by a salt bridge with D73. Ligand/sidechain oxygen, nitrogen and phosphorus atoms are coloured red, blue and orange, respectively. Bond distances are given in Å to 1 decimal place. **d**, Multiple sequence alignment of fully annotated DNPH1 protein sequences (N-terminal halves only). Symbols underneath each row indicate consensus level. '*' indicates a single, fully conserved residue; ':' indicates conservation of residues with strongly similar properties; '.' indicates conservation of residues with weakly similar properties. R30-D73 salt bridge residues are indicated by green arrows. Ligand-binding residue H56 is indicated by a red arrow.

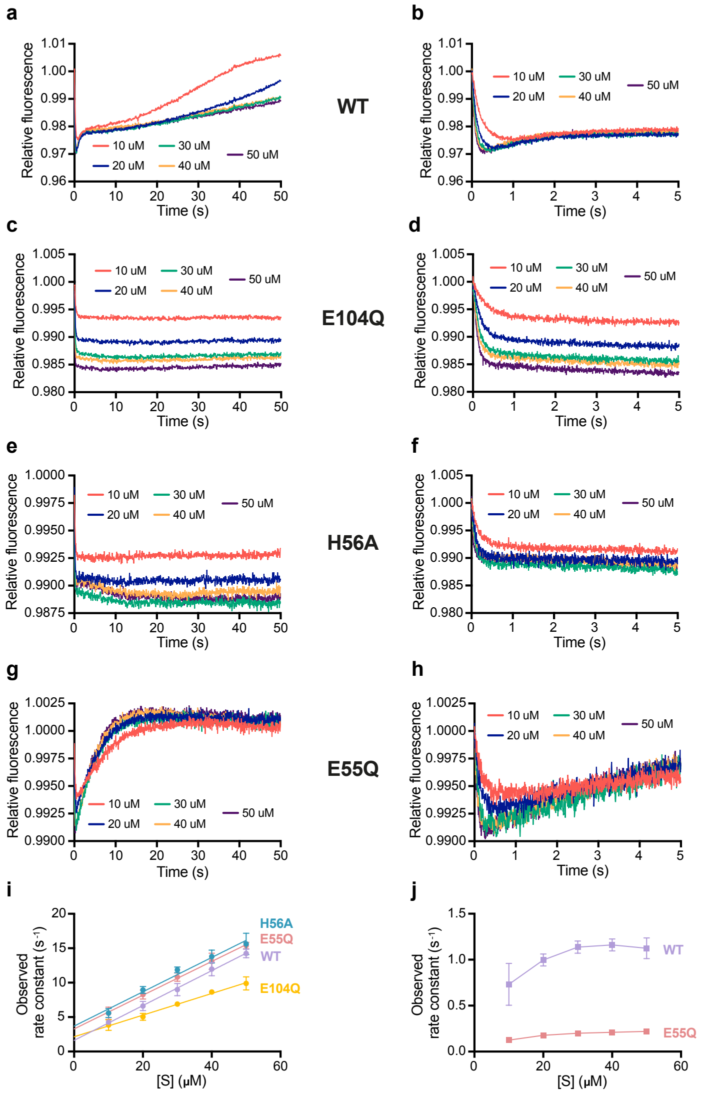

Supplementary Fig. 4: Stopped-flow data and analyses for DNPH1 point mutants clarify catalytic residue function. a – h, Normalised stopped-flow measurements of tryptophan fluorescence during substrate binding/hydrolysis by DNPH1^{WT} (a and b), DNPH1^{E104Q} (c and d), DNPH1^{H56A} (e and f) and DNPH1^{E55Q} (g and h). Representative plots of single datasets are averages of three technical repeats for 2 μM enzyme and various substrate concentrations; each experiment was independently repeated at least three times. a, c, e and g, Long timescale data are corrected for fluorescence quenching. b, d, f and h, Short timescale data are not corrected for fluorescence quenching. **i**, Plot of primary observed rate constant vs [hmdUMP] for DNPH1 point mutants. Data points and error bars represent the mean and standard deviation, respectively, from n=3 (WT, E104Q) or n=4 (H56A, E55Q) independent experiments. Gradients and Y-intercepts for lines of best-fit determine k_1 and k_{-1} rate constants respectively, yielding a substrate dissociation constant (K_D) for each enzyme. **j**, Plot of secondary observed rate constant vs [hmdUMP] for DNPH1^{WT} and DNPH1^{E55Q}. Data points and error bars represent the mean and standard deviation, respectively, from n=3 (WT) or n=4 (E55Q) independent experiments. For each dataset, the mean value at the point of plateau provides an estimate for the rate constant k_2. Source data are provided as a Source Data file.

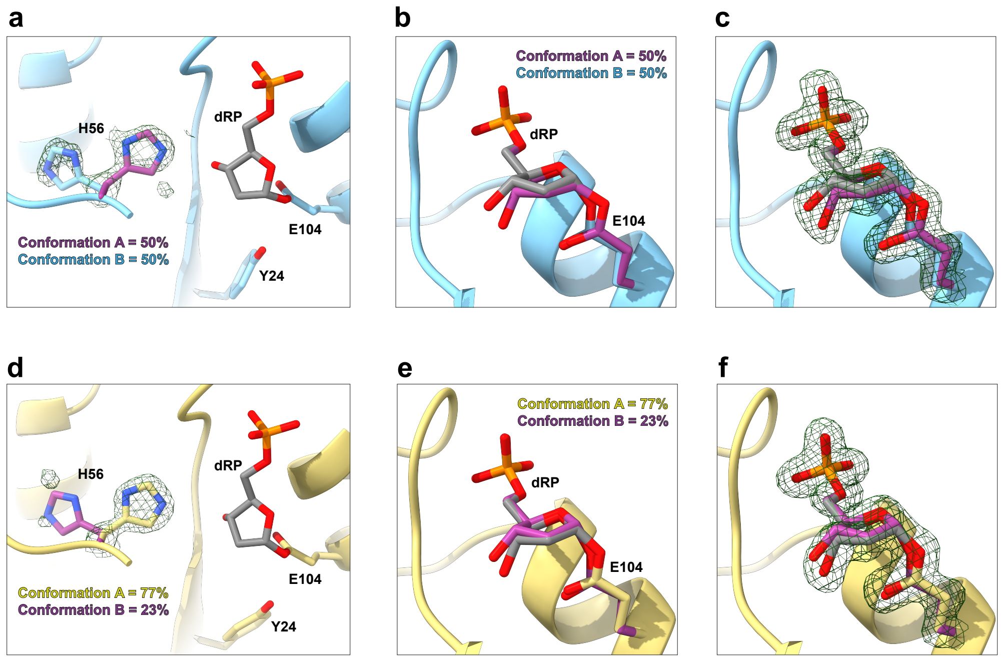

Supplementary Fig. 5: H56 and fused dRP ligand adopt two conformations in each DNPH1^{E55Q} active site. a – **c**, Chain A (blue) with fused dRP (grey sticks). In a, H56 rotamers 'A' (magenta) and 'B' (blue) are shown in the context of dRP:E104 conformation 'B'. Electron density map (green mesh) contoured to 1 σ around H56 sidechains. In b, the fused dRP conformation 'B' is in grey, and the dRP:E104 conformation 'A' is shown in magenta. Panel c, as b, but with the electron density map (green mesh) contoured to 1.5 σ around the fused dRP and E104 sidechain. **d** – **f**, Chain B (yellow) with fused dRP (grey sticks). In d, the H56 rotamers 'A' (yellow) and 'B' (magenta) are shown in the context of dRP:E104 conformation 'A'. Electron density map (green mesh) is contoured to 1.5 σ around the H56 sidechains. In e, the fused dRP conformation 'A' is shown in grey, and the dRP:E104 conformation 'B' is in magenta. f, as e, with electron density map (green mesh) contoured to 1.5 σ around the fused dRP and E104 sidechain. Ligand/sidechain oxygen, nitrogen and phosphorus atoms are coloured red, blue and orange, respectively. Nearby waters are omitted for clarity. Colour-coded percentages refer to refined occupancies for the indicated rotamers/conformations.

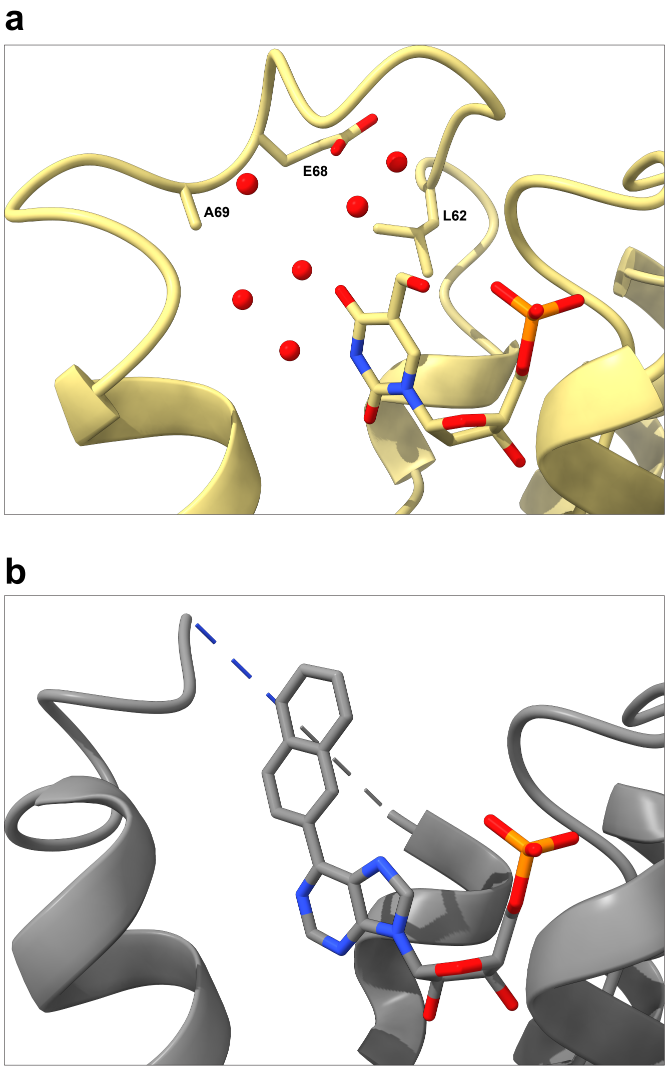

Supplementary Fig. 6: The DNPH1 shielding loop traps water around the base. **a**, DNPH1^{E104Q} chain B bound to hmdUMP (yellow), highlighting water molecules (red spheres) encapsulated by the shielding loop. Ligand/sidechain oxygen, nitrogen and phosphorus atoms are coloured red, blue and orange, respectively. **b**, chain A of DNPH1 bound to a purine-based inhibitor (grey; PDB 4P5E), highlighting overlap of ligand with shielding loop and bound water visualised in (a).

Supplementary Table 1: Diffraction data and model refinement statistics.

	DNPH1^{E104Q}	DNPH1^{E55Q}
**Data collection***		
Space group	P 1 2_1 1	P 2_1 2_1 2_1
Cell dimensions		
a, *b*, *c* (Å)	56.648, 67.126, 120.117	47.42, 55.05, 103.98
α, β, γ (°)	90.000, 99.633, 90.00	90.00, 90.00, 90.00
Resolution (Å)	59.212-1.775 (1.806-1.775)	103.98-1.65 (1.68-1.65)
R_{sym} or R_{merge}	0.125 (2.152)	0.258 (3.982)
R_{meas}	0.132 (2.418)	0.293 (4.512)
R_{pim}	0.042 (1.059)	0.137 (2.096)
$CC_{1/2}$	0.999 (0.398)	0.994 (0.164)
$I / \sigma I$	11.7 (0.7)	5.6 (0.5)
Completeness (%)	93.5 (77.9)	99.8 (99.1)
Redundancy	9.5 (4.6)	8.4 (8.6)
Wilson B factor (Å^2)	25.79	19.12
**Refinement**		
Resolution (Å)	55.85-1.78	43.14-1.65
No. reflections	79,801	33372
R_{work} / R_{free}[a]	0.2056 / 0.2245	0.1845 / 0.2218
No. of non-hydrogen atoms	7493	2563
Protein	6830	2198
Ligand/ion	164	75
Water	499	290
B-factors (Å^2)	37.19	23.83
Protein	37.18	22.57
Ligand/ion	45.71	29.99
Water	39.28	34.06
R.m.s. deviations		
Bond lengths (Å)	0.0021	0.0032
Bond angles (°)	0.558	0.681
Ramachandran plot		
Favored (%)	98.15%	97.25
Allowed (%)	1.85%	2.75
MolProbity score	0.81	0.64

**Values in parentheses are for highest-resolution shell.*

[a]5% of reflections were randomly selected for calculating R_{free}.

Supplementary Table 2: Observed rate constants and affinities for DNPH1 point mutants.

	k_1 ($\mu M^{-1}.s^{-1}$)	k_{-1} (s^{-1})	K_D (μM)	k_2 (s^{-1})	k_3 (s^{-1})
**$DNPH1^{WT}$**	0.25 ± 0.01	1.6 ± 0.4	6.4 ± 1.9	~1.2*	~0.15^
**$DNPH1^{E104Q}$**	0.16 ± 0.01	2.2 ± 0.4	14± 3	ND	ND
**$DNPH1^{H56A}$**	0.25 ± 0.02	3.6 ± 0.5	14 ± 3	~0.01^	ND
**$DNPH1^{E55Q}$**	0.25 ± 0.01	3.3 ± 0.4	13 ± 2	~0.22*	~0.001^

* Calculated from stopped-flow analysis.
^ Calculated from steady-state rate.

Supplementary Table 3: Crystallization and cryoprotection conditions for diffracting crystal samples.

	**DNPH1^{E104Q}**	**DNPH1^{E55Q}**
Crystallization buffer	0.2 M sodium fluoride<br>0.1 M Bis-Tris propane pH 6.5<br>20% w/v PEG 3350	0.1 M Bis-Tris propane pH 6.5<br>16% w/v PEG 3350
Cryoprotectant	0.2 M sodium fluoride<br>0.1 M Bis-Tris propane pH 6.5<br>20% w/v PEG 3350<br>30% v/v ethylene glycol	0.1 M Bis-Tris propane pH 6.5<br>20 mM HEPES pH 7.5<br>0.5 mM TCEP<br>0.1 M sodium chloride<br>15% w/v PEG 3350<br>25% v/v ethylene glycol
