# Supplementary material for: Mechanism of substrate hydrolysis by the human nucleotide pool sanitiser DNPH1
Source: Nat Commun. 2023 Oct 26;14:6809. doi: 10.1038/s41467-023-42544-4 (PMC10603095; doi:10.1038/s41467-023-42544-4)
Supplement: Supplementary file 3 — Reporting Summary [file 41467_2023_42544_MOESM3_ESM.pdf]

nature portfolio

Corresponding author(s): West, S.C.

Last updated by author(s): Oct 11, 2023

Reporting Summary

Nature Portfolio wishes to improve the reproducibility of the work that we publish. This form provides structure for consistency and transparency in reporting. For further information on Nature Portfolio policies, see our Editorial Policies and the Editorial Policy Checklist.

Statistics

For all statistical analyses, confirm that the following items are present in the figure legend, table legend, main text, or Methods section.

n/a	Confirmed	
☐	☒	The exact sample size (*n*) for each experimental group/condition, given as a discrete number and unit of measurement
☐	☒	A statement on whether measurements were taken from distinct samples or whether the same sample was measured repeatedly
☒	☐	The statistical test(s) used AND whether they are one- or two-sided<br>*Only common tests should be described solely by name; describe more complex techniques in the Methods section.*
☒	☐	A description of all covariates tested
☒	☐	A description of any assumptions or corrections, such as tests of normality and adjustment for multiple comparisons
☐	☒	A full description of the statistical parameters including central tendency (e.g. means) or other basic estimates (e.g. regression coefficient) AND variation (e.g. standard deviation) or associated estimates of uncertainty (e.g. confidence intervals)
☒	☐	For null hypothesis testing, the test statistic (e.g. *F*, *t*, *r*) with confidence intervals, effect sizes, degrees of freedom and *P* value noted<br>*Give P values as exact values whenever suitable.*
☒	☐	For Bayesian analysis, information on the choice of priors and Markov chain Monte Carlo settings
☒	☐	For hierarchical and complex designs, identification of the appropriate level for tests and full reporting of outcomes
☒	☐	Estimates of effect sizes (e.g. Cohen's *d*, Pearson's *r*), indicating how they were calculated

Our web collection on statistics for biologists contains articles on many of the points above.

Software and code

Policy information about availability of computer code

Data collection	Commercial software available with the respective instruments was used for data collection. These include UNICORN 7.3 (Cytiva) for protein purification, General Data Acquisition ('GDA', OpenGDA) software for X-ray crystallography data collection, Chromnav software (v 1.19, Jasco) for HPLC analyses, SpectraManager (v 2.5, Jasco) for protein spectrophotometry.
Data analysis	X-ray crystallography data were analysed and processed using either AutoPROC (Global Phasing, DNPH1-E104Q only) or DUI/DIALS and POINTLESS/AIMLESS as part if the CCP4/CCP4i2 package (DNPH1-E55Q only). Anisotropy and eventual spherical high-resolution cutoff was determined by STARANISO (Global Phasing, DNPH1-E55Q only).<br>For both X-ray crystallography datasets, molecular replacement was performed using Phaser as part of the Phenix software package (v 1.20.1-4487). Molecular models were manually corrected and refined using Coot (v 0.9.8.7) and phenix.refine respectively. Validation was performed using MolProbity. Model visualisation was performed using ChimeraX (v 1.4).<br>ClustalOmega was used for multiple sequence alignment.<br>PDBSum was used for structural topology analysis.<br>ConSurf was used for 3D-conservation analysis.<br>LigPlot+ was used for ligand interaction analyses.<br>GraphPad Prism 9 (v 9.2) was used for statistical analysis and data plotting<br>Kinetic Studio 5 (TgK Scientific Ltd) was used for stopped-flow data analysis<br>Xcalibur Qual Browser and Tracefinder 4.1 software (Thermo Scientific) were used for analysis of mass spectrometry data

For manuscripts utilizing custom algorithms or software that are central to the research but not yet described in published literature, software must be made available to editors and reviewers. We strongly encourage code deposition in a community repository (e.g. GitHub). See the Nature Portfolio guidelines for submitting code & software for further information.

Data

Policy information about availability of data

All manuscripts must include a data availability statement. This statement should provide the following information, where applicable:

- Accession codes, unique identifiers, or web links for publicly available datasets
- A description of any restrictions on data availability
- For clinical datasets or third party data, please ensure that the statement adheres to our policy

The datasets generated during and/or analysed during the current study are included alongside the Article or are available from S.C.W. on reasonable request. Comparisons were made to inhibitor-bound hDNPH1 and MilB, which are available in the PDB under the accession codes 4P5E and 4OHB respectively. Structure factor files and and atomic models of DNPH1-E104Q and DNPH1-E55Q have been deposited in the Protein Database (PDB) under accession codes 8QHQ and 8QHR respectively. All other data is archived at the Francis Crick Institute.

Research involving human participants, their data, or biological material

Policy information about studies with human participants or human data. See also policy information about sex, gender (identity/presentation), and sexual orientation and race, ethnicity and racism.

Reporting on sex and gender	N/A
Reporting on race, ethnicity, or other socially relevant groupings	N/A
Population characteristics	N/A
Recruitment	N/A
Ethics oversight	N/A

Note that full information on the approval of the study protocol must also be provided in the manuscript.

Field-specific reporting

Please select the one below that is the best fit for your research. If you are not sure, read the appropriate sections before making your selection.

☒ Life sciences ☐ Behavioural & social sciences ☐ Ecological, evolutionary & environmental sciences

For a reference copy of the document with all sections, see nature.com/documents/nr-reporting-summary-flat.pdf

Life sciences study design

All studies must disclose on these points even when the disclosure is negative.

Sample size	No statistical analyses were performed to predetermine sample size. Wherever quantification is provided for biochemistry experiments, a minimum of 3 independent experiments (as per standard practices) were carried out to allow statistical analyses to be performed. The exception to this is HPLC-based substrate turnover assays, which were performed as 2 independent experiments, with a minimum of 4 different time points. HPLC data for WT DNPH1 were verified against previously published work (Fugger, K. et al., 2021) to ensure internal consistency.
Data exclusions	In general, no data was excluded except where experiments failed due to technical problems. During X-ray data processing and model refinement, automated outlier rejection algorithms as part of software packages were utilised with default settings, as is standard practice.
Replication	Biochemical experiments were performed in triplicate on separate days to ensure reproducibility. The exception to this is HPLC-based substrate turnover assays, which were performed as 2 independent experiments, with a minimum of 4 different time points. Barring technical issues, all replication attempts were successful. Crystallographic experiments were not replicated - this is standard practice.
Randomization	To enable bias-free analysis of model-to-data agreement in X-ray crystallography experiments, a random set of ~5% of the data were excluded from model refinement. This 'FreeR' set was assigned automatically by the described software packages, as is standard practice. Biochemical experiments did not make use of experimental groups or randomization as these are not appropriate.
Blinding	Blinding is not necessary for analysis of recombinant proteins by X-ray crystallography or biochemistry. The researcher needs to know the protein sample used to conduct the experiments performed, and therefore blinding is not appropriate.

Reporting for specific materials, systems and methods

We require information from authors about some types of materials, experimental systems and methods used in many studies. Here, indicate whether each material, system or method listed is relevant to your study. If you are not sure if a list item applies to your research, read the appropriate section before selecting a response.

Materials & experimental systems

n/a	Involved in the study
☒	☐ Antibodies
☒	☐ Eukaryotic cell lines
☒	☐ Palaeontology and archaeology
☒	☐ Animals and other organisms
☒	☐ Clinical data
☒	☐ Dual use research of concern
☒	☐ Plants

Methods

n/a	Involved in the study
☒	☐ ChIP-seq
☒	☐ Flow cytometry
☒	☐ MRI-based neuroimaging
